# Polyethylene Glycol 3350 in the Treatment of Chronic Idiopathic Constipation: Post hoc Analysis Using FDA Endpoints

**DOI:** 10.1155/2022/3533504

**Published:** 2022-09-09

**Authors:** Stacy B. Menees, Anthony J. Lembo, William D. Chey

**Affiliations:** ^1^Division of Gastroenterology & Hepatology, Michigan Medicine, Ann Arbor, MI, USA; ^2^Division of Gastroenterology, Department of Internal Medicine, Ann Arbor Veterans Affairs Medical Center, Ann Arbor, Michigan, USA; ^3^Division of Gastroenterology, Beth Israel Deaconess Medical Center, Boston, MA, USA; ^4^Division of Gastroenterology, Michigan Medicine, Ann Arbor, MI, USA

## Abstract

**Methods:**

This multicenter, double-blind, placebo-controlled, parallel-group trial included adults with chronic idiopathic constipation randomized to polyethylene glycol 3350 17 g (*n* = 204) or placebo (*n* = 100) once daily for 24 weeks. Post hoc analyses were performed using the US Food and Drug Administration endpoint (≥3 complete spontaneous bowel movements/week and an increase of ≥1 complete spontaneous bowel movement/week from baseline for ≥9/12 weeks, including 3 of the last 4 weeks) along with additional efficacy and safety outcomes.

**Results:**

The proportion of patients meeting the new endpoint was significantly higher with polyethylene glycol 3350 vs placebo (42% vs 13%; *P* < 0.0001). Reductions in the mean number of hard/lumpy stools/week (–2.1 vs –0.9; *P* = 0.0014) and the weekly mean five-point cramping rating (–0.3 vs –0.1; *P* = 0.0272) also significantly favored polyethylene glycol 3350. The proportion of subjects with gastrointestinal adverse events decreased markedly after the first week of treatment in the polyethylene glycol 3350 group.

**Conclusion:**

Using the current US Food and Drug Administration-recommended responder definition and other secondary outcomes, once-daily polyethylene glycol 3350 demonstrated substantial and sustained efficacy and safety over 24 weeks in patients with chronic idiopathic constipation. *Trial Registration.* The original trial was registered with https://clinicaltrials.gov Trial: NCT00153153.

## 1. Introduction

Chronic constipation is a common multi-symptom condition in the general population with self-reported prevalence estimates ranging from 5% to 27% [1]. This is generally higher than the prevalence of chronic constipation defined by Rome criteria, with one systematic review placed between 6.8% and 15% [2]. Associated symptoms include hard/lumpy stools, abdominal discomfort, bloating/distension, straining, and feelings of incomplete evacuation [3]. Although many over-the-counter (OTC) agents are available for the treatment of constipation, the majority have very limited published efficacy data, and studies that have been conducted used non-standardized endpoints with short durations of treatment [4].

Polyethylene glycol (PEG) 3350 is an osmotic laxative that initially received US Food and Drug Administration (FDA) approval as a prescription product for the treatment of occasional constipation in 1999 and subsequently received approval for OTC use in 2006 under the brand name MiraLAX® (Bayer HealthCare LLC, Whippany, NJ). The efficacy and safety of PEG 3350 have been demonstrated in numerous randomized controlled trials vs placebo [5–9] and other agents [10,11]. However, since the FDA approval of PEG 3350, new clinical standards and endpoints for the assessment of constipation therapies have been adopted. Beginning in the early 2010s, a new primary efficacy endpoint (“FDA chronic idiopathic constipation (CIC) endpoint”) of at least three complete spontaneous bowel movements (CSBMs) per week and an increase of at least one CSBM/week from baseline for at least 9 weeks of the 12-week treatment period was commonly utilized in chronic constipation studies of prescription therapies [12–16]. Given the lack of evidence assessing response to OTC laxative therapy using modern, widely accepted endpoints, we were interested in reanalyzing previously generated study data using the FDA-recommended endpoint, which is more relevant to today's clinicians and researchers. Thus, this post hoc analysis aims to reevaluate data from a previously published 6-month placebo-controlled trial [5] using the current FDA CIC endpoint.

## 2. Materials and Methods

### 2.1. Study Design

The study design has been previously described (NCT00153153) [5]. This was a randomized, double-blind, placebo-controlled, parallel-group, 50-center trial that included adults with CIC based on modified Rome I criteria: less than three satisfactory bowel movements (BMs) per week for the preceding 3 months when not taking laxatives plus at least one of the three remaining symptom criteria: (1) straining in more than 25% of defecations; (2) lumpy or hard stools in more than 25% of defecations; and (3) a sensation of incomplete evacuation in more than 25% of defecations. Subjects were also required to have less than three satisfactory BMs per week during the 14-day baseline pretreatment observation period. Subjects were randomized (2 : 1) to receive PEG 3350 17 g or placebo once daily for 6 months (24 weeks). Rescue medication with bisacodyl 10 mg was allowed for those who experienced severe constipation-related discomfort or had no BMs for 4 days.

### 2.2. Outcomes

Bowel movement experiences and safety data were collected daily using an Interactive Voice Response System (Interactive Clinical Technologies, Inc., Yardley, PA). Upon calling in, patients were asked to report the number and characteristics of the BMs. This included whether the BMs were complete, were satisfactory, or were lumpy or hard; whether straining was required to pass the stool; and whether laxatives were used. Patients also rated the amount of cramping and the amount of gas each day. In the original analysis, the primary endpoint was treatment success based on an assessment of modified Rome criteria for each treatment week. A weekly CSBM response is defined as at least three CSBMs per week and an increase of at least one CSBM from baseline. The FDA CIC endpoint is defined as a weekly CSBM response for at least 9 of 12 treatment weeks, including 3 of the last 4 weeks of the treatment period. For purposes of this study, the CIC endpoint applies to the initial 12-week period and separately for the latter 12-week period of the full 24-week study. These criteria were further expanded to assess weekly CSBM response across additional time intervals throughout the 6-month study, including the weekly CSBM responder rates for at least 18 of 24 weeks (i.e., 18/24 endpoint) and the less stringent endpoints of response for at least 6 of 12 weeks (i.e., 6/12 endpoint) and for at least 12 of 24 weeks (i.e., 12/24 endpoint). Additionally, the data were analyzed using a more stringent endpoint, where the overall sustained CSBM responders were those who met the CSBM weekly responder definition for at least 9 of the first 12 weeks *and* at least 9 of the last 12 weeks of treatment. All analyses were also conducted for spontaneous bowel movement (SBM) response.

To further assess the effect of PEG 3350 on individual constipation-related symptoms, additional efficacy analyses were performed to assess the mean number of BMs with straining and hard/lumpy stools as well as mean qualitative scores for cramping and gas. Cramping and gas severity was assessed by subjects on a 5-point scale from zero (symptom absent) to four (symptom is extreme). Symptoms were recorded daily, and assessments of these endpoints were performed over the entire 24-week study period and for each individual week.

### 2.3. Safety

As general safety and tolerability findings were described in the original analysis, this safety analysis focused specifically on the time course of gastrointestinal adverse events (GI AEs). This analysis evaluated (1) the proportion of subjects with treatment-emergent GI AEs in the first 12 weeks vs the last 12 weeks and (2) the proportion of subjects with treatment-emergent GI AEs in each distinct week of the 6-month treatment period. In addition, the proportion of patients with investigator-defined drug-related GI AEs was determined. This included AEs with a relationship to the study drug defined as possibly related, probably related, definitely related, or AEs for which assessment of the relation to the study drug was not available.

### 2.4. Statistical Analyses

Statistical comparisons for CSBM and SBM outcomes were performed using the Cochran–Mantel–Haenszel chi-squared test. An analysis of variance with treatment, pooled-site, and their interaction as covariates was used to compare the effects of PEG 3350 and placebo on constipation-associated symptoms (i.e., straining, hard/lumpy stools, cramping, and gas). Adverse events were compared between treatment groups using a two-tailed Fisher's exact test. All randomized subjects who took at least one dose of PEG 3350 or placebo were included in the intention-to-treat analysis.

All authors had access to the data from this post hoc analysis and had reviewed and approved the final manuscript.

## 3. Results

### 3.1. Subjects

There were 304 subjects in the intention-to-treat population (204 PEG and 100 placebo). Of these, 170 subjects completed all 6 months of the study (Supplemental Figure 1). Baseline demographic and disease characteristics were generally similar between treatment groups (Table 1). The mean age was 53 years (range 20–92 years), and 75 were 65 years or older. The majority of patients were female (85%) and Caucasian (83%). The mean duration of constipation was 23 years. At baseline, patients experienced an average of fewer than one CSBM and four or fewer SBMs per week in both treatment groups. At baseline, the mean number of CSBMs and SBMs were fewer than one per week and four or fewer per week, respectively, in both treatment groups (Table 1).

### 3.2. Efficacy

#### 3.2.1. FDA CIC Endpoint

**Figure 1:**
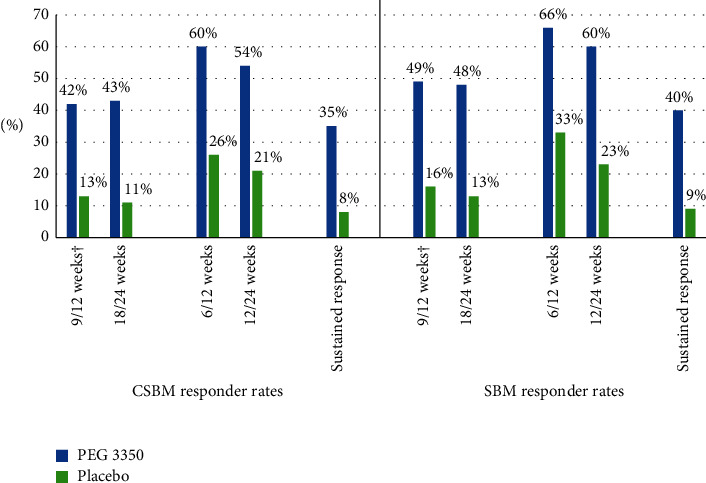
CSBM and SBM response rates using different time intervals. Abbreviations: CIC, chronic idiopathic constipation; CMH, Cochran–Mantel–Haenszel; CSBM, complete spontaneous bowel movement; FDA, US Food and Drug Administration; PEG, polyethylene glycol; SBM, spontaneous bowel movement. ^†^FDA CIC endpoint: weekly responder for ≥9 weeks out of the 12-week treatment period including 3 of the last 4 weeks of the period. *P* < 0.0001 for PEG 3350 vs placebo for all CSBM/SBM responder definitions. *P* value obtained from CMH chi-squared test, adjusting for pooled study sites, to ensure homogeneity/consistency across the treatment arms. A weekly CSBM/SBM responder is a patient who has at least three CSBMs/SBMs and at least one CSBM/SBM more than baseline in a week, and data are available on at least 4 days in a respective week. A continuous CSBM/SBM responder is a responder for ≥9 out of the first 12 weeks and ≥9 out of the last 12 weeks.

A significantly higher proportion of patients were FDA CIC endpoint responders in the PEG 3350 group compared to those receiving placebo (42% vs 13%; *P* < 0.0001; Figure 1 and Supplemental Table 1).

#### 3.2.2. CSBM/SBM Responses

**Figure 2:**
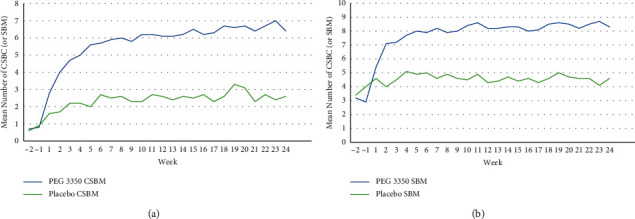
Mean weekly CSBMs (a) and SBMs (b) in subjects receiving PEG 3350 or placebo. †The change from baseline in the mean number of SBM and the mean number of CSBM each week was significantly greater with PEG 3350 versus placebo at week 1 (*P*=0.0040 and *P*=0.0013, respectively) and highly significantly greater (*P* < 0.0001 for both outcomes) each week for weeks 2 through 24. Abbreviations: CSBM, complete spontaneous bowel movement; PEG, polyethylene glycol; SBM, spontaneous bowel movement.

After the initiation of treatment, the mean number of CSBMs (Figure 2(a)) and SBMs (Figure 2(b)) per week increased rapidly in the PEG 3350 group, leveling out after 4 to 8 weeks with frequencies ranging from 6 to 7 per week and 8 to 9 per week, respectively, over the 24-week study duration. By contrast, mean CSBMs and SBMs ranged from 2 to 3 per week and 4 to 5 per week, respectively, for those receiving placebo over the 24-week treatment period.

Complete spontaneous bowel movement and SBM responses for other time points are summarized in Figure 1 and Supplemental Table 1. Response remained consistent for the 18-/24-week analysis, with CSBM response observed in 43% of patients in the PEG 3350 group and 11% of those receiving placebo (*P* < 0.0001). Similar results were seen for SBM responders at both the 9-/12-week (49% vs 16%; *P* < 0.0001) and 18-/24-week (48% vs 13%; *P* < 0.0001) time points. Complete spontaneous bowel movement and SBM responses were also significantly greater than placebo (*P* < 0.0001) when the less stringent 6-/12-week and 12-/24-week endpoints were used (Figure 1; Supplemental Table 1).

The proportion of overall sustained CSBM and SBM responders (i.e., responders for ≥9 out of the first 12 weeks *and* ≥9 of 12 out of the last 12 weeks of treatment) was significantly greater for PEG 3350 than placebo (35% vs 8%; *P* < 0.0001 for CSBM response and 40% vs 9%; *P* < 0.0001 for SBM response).

Overall, PEG 3350-treated patients used fewer tablets of rescue medication (bisacodyl) on average than placebo-treated patients; however, this difference (2.85 versus 3.90 tablets per week, respectively) did not reach statistical significance (*P*=0.138). Half of the PEG 3350 study patients used ≤8 bisacodyl tablets over the 6-month treatment period, and approximately 21% in the PEG 3350 group did not use any bisacodyl during the study treatment.

#### 3.2.3. Constipation-Associated Symptoms

**Figure 3:**
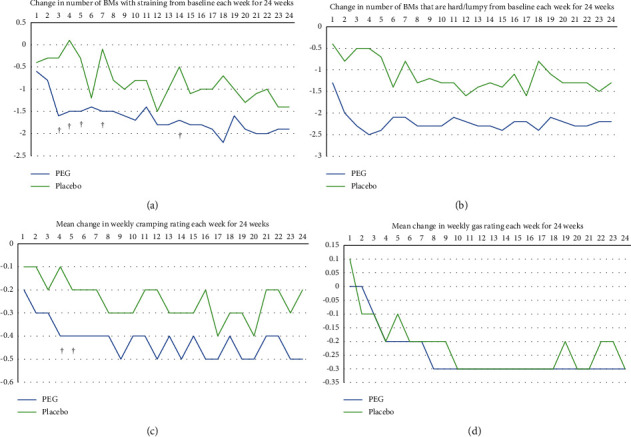
Mean change in the number of BMs associated with straining (a), hard/lumpy stools (b), cramping (c), and gas (d) over time. Abbreviations: BM, bowel movement; PEG, polyethylene glycol. ^†^*P* < 0.05. Baseline is defined as the average of 2 weeks before treatment (i.e., weeks −2 and −1). Analysis based on available non-missing values in each week. *P* value derived from the analysis of variance with treatment, pooled-site, and their interaction as covariates.

The mean weekly change from baseline over 24 weeks in constipation-associated symptoms (i.e., straining, hard/lumpy stools, cramping, and gas) is summarized in Table 2. A significantly greater reduction in the mean number of hard/lumpy stools per week (–2.1 vs –0.9; *P*=0.0014) and in the weekly mean five-point cramping rating (–0.3 vs –0.1; *P*=0.0272) was observed in the PEG 3350 group compared with placebo-treated subjects over the 24-week study period. The mean reductions in the number of BMs with straining per week (–1.4 vs –0.8; *P*=0.0799) and in the five-point gas rating (–0.1 vs –0.1; *P*=0.5949) were not significantly different between treatment groups for the overall treatment period, though the change in the number of BMs with straining tended to favor PEG 3350. Furthermore, PEG 3350 was associated with significant improvements in straining, hard/lumpy stools, and cramping at several individual weekly time points than placebo. There was no difference in gas severity between PEG 3350 and placebo at any time point over 24 weeks of treatment (Figure 3).

### 3.3. Safety

In the original published study, it was reported that subjects in the PEG 3350 group experienced significantly more GI complaints than placebo (39.7% vs 25%; *P* = 0.015) throughout the study, with the difference driven by abdominal distension, diarrhea, loose stools, flatulence, and nausea [5].  Table 3 shows the updated analysis with rates of GI AEs that occurred in at least 2% of any treatment group broken down by the first and second halves of the study (i.e., weeks 1–12 vs. weeks 13–24). During weeks 1 through 12, significantly more subjects receiving PEG 3350 experienced at least one GI AE compared to the placebo group (34% vs. 17%; *P* = 0.0018). The difference was primarily driven by diarrhea (14% vs. 6%; *P* = 0.0525) and loose stools (8% vs. 1%;*P* = 0.0148). However, during weeks 13 through 24, there was no significant difference between treatment groups for the number of subjects with at least one GI AE (9% vs. 12%; *P* = 0.5455) or for any individual AE. Overall, the proportion of subjects with at least one GI AE decreased from 34% in weeks 1 through 12 to 9% in weeks 13 through 24 in the PEG 3350 group. A corresponding attenuation of AEs over time in the placebo group was not observed, with at least one GI AE occurring in 17% of patients assigned to placebo during weeks 1 through 12 and 12% of patients assigned to placebo during weeks 13 through 24. Supporting these findings, the analysis of the GI AEs by week showed that the number of subjects with AEs decreased markedly after the first week of treatment. The proportion of subjects in the PEG 3350 group with at least one GI AE decreased from 12% in week 1 to 7% in weeks 2 and 3 and did not increase to above 4% for the remainder of the 24-week study period. In the placebo group, the GI AE prevalence decreased from 8% in week 1 to 2% in week 2.

Similar results were observed when AEs were assessed by the investigator to be related to treatment. From weeks 1 through 12 to weeks 13 through 24, the percentage of patients with at least one AE judged as related to the intervention was reduced from 29% during weeks 1 through 12 to 7% during weeks 13 through 24 with PEG 3350. The change observed with placebo was less dramatic (13% during weeks 1–12 to 9% during weeks 13–24). Although significantly more investigator-defined AEs were observed during weeks 1 through 12 with PEG 3350 vs placebo (29% vs 13%; *P*=0.0024), there was no significant difference in the frequency of AEs during weeks 13 through 24 (7% vs 9%; *P*=0.4976).

Most AEs were mild-to-moderate in severity. There were six serious AEs experienced by four PEG-treated subjects, while six serious AEs occurred in five subjects in the placebo group. None of the serious AEs in either group were considered related to study medication by the investigators. Nineteen of 204 PEG 3350-treated subjects (9.3%) and 7 of 100 placebo-treated subjects (7%) withdrew because of an AE. The treatment-related discontinuations were primarily related to GI events, consistent with and expected from laxative use. Withdrawal due to lack of efficacy occurred in 24 of 204 (11.8%) and 26 of 100 (26%) subjects, respectively, in the PEG 330 and placebo groups.

## 4. Discussion

Studies evaluating most OTC preparations for the management of constipation are limited and of variable quality [4]. The most commonly used outcome measures in these studies were stool frequency (i.e., CSBMs, SBMs, or BMs), stool consistency, and treatment response, but how these were defined and measured and the intervals of measurement varied considerably between studies. Given the lack of a standardized efficacy outcome measure, it is difficult to assess the relative efficacy of available agents [4]. Although there is a considerable amount of high-quality clinical evidence supporting the use of PEG 3350 in constipation [5–7,11], these studies used response criteria that are less relevant to today's clinicians and researchers. This is the first analysis evaluating the efficacy of PEG 3350 (or any OTC constipation therapy) in patients with CIC using the current, unmodified FDA weekly responder definition evaluated over 12 weeks. The goal was to assess the efficacy and safety of PEG 3350 in a clinically meaningful manner that would also facilitate, admittedly imperfect, comparisons to other constipation therapies. In this current analysis that reassesses a previously published placebo-controlled trial, CSBM and SBM increased rapidly in the PEG 3350 group and remained significantly improved throughout the 24-week study duration. Using current definitions of treatment response based on the FDA CIC endpoint, the response was achieved in 42% of patients receiving PEG 3350 vs 13% of placebo patients. Sustained efficacy of PEG 3350 was also demonstrated over the 6-month treatment period.

Although other OTC treatments for CIC have not been evaluated using these criteria, the response rate achieved in this analysis (i.e., 42% for PEG 3350) compares favorably to response rates observed with other prescription therapies that have been studied using the FDA-recommended criteria (Supplemental Table 2) [12–18]. For example, studies evaluating linaclotide have reported response rates of 12% to 21% using the definition of at least three CSBMs per week and an increase of at least one CSBM per week from baseline for at least 9 of 12 weeks [12–14]. Using this same definition but adding a criterion for durability (i.e., positive response 3 of the last 4 weeks), plecanatide has been associated with CSBM responses of 20% to 21% [15,16]. Studies evaluating prucalopride have reported response rates of 25% to 38% using the less stringent definition of achieving at least three CSBMs per week for 12 to 24 weeks [17,18] with one study showing no significant difference vs placebo [18].

Notably, similar results were observed in our analysis when different responder definitions were used. This includes the more stringent overall sustained CSBM response criteria (i.e., those who met the FDA CSBM weekly responder definition for ≥9 out of the first 12 weeks *and* ≥9 out of the last 12 weeks) as well as the less stringent 6-/12-week (i.e., weekly response in ≥6 of 12 weeks) and 12-/24-week (i.e., weekly response in ≥12 of 24 weeks) endpoints. The significant efficacy across multiple time intervals for both CSBM and SBM and different definitions demonstrates the robustness of the findings and overall consistency in the clinical benefits of PEG 3350 for subjects with CIC. PEG 3350 was also associated with significant improvements in some constipation-related symptoms (i.e., number of hard/lumpy stools, subject-rated cramping), with no difference between groups for subject-rated symptoms of gas. These findings are important given the multi-symptom nature of constipation.

Readers need to exercise caution when trying to directly compare results from different trials. Though our post hoc analyses harmonized the main outcome assessments, important differences between trials remain. For example, our study with PEG 3350 enrolled patients who fulfilled modified Rome I criteria as opposed to the more restrictive Rome III or IV criteria in more recent studies. In addition, these studies were done at different points in time. It is difficult to know whether the gradual increase in the availability of OTC and prescription treatment options could have been selected for constipated patients who had failed multiple therapies in some of the more recent trials. It is reassuring that study population demographics and baseline CSBM rates were similar across the studies.

The safety analyses provide insight into the time course of AEs with long-term PEG 3350 therapy. Although GI AEs were significantly more common than placebo in the first 12 weeks of treatment, there was no significant difference between the PEG 3350 and placebo groups in the second 12 weeks. Further, the weekly analyses showed a substantial drop in AEs after the first week of treatment followed by a sustained low rate of AEs as patients continued therapy. Overall, these findings provide reassurance that PEG 3350-related AEs are often transient and that the agent is well tolerated with both short- and long-term therapy.

A limitation of this study includes the post hoc nature of the analysis. Further, how BM data were collected could have influenced the results. Patients were asked to self-report the number of BMs they had each day and were then asked to specify whether the BMs for the day were complete or not. In situations where someone had an SBM and CSBM on the same day, they could have answered in either direction regarding whether their BM was complete. Additionally, there was a high number of dropouts during the study (57% and 38%, respectively, in the placebo and PEG 3350 groups). This is higher than that reported in trials for both active and placebo treatment in 12-week trials evaluating other agents [13–18]. This may be at least partially related to the longer (i.e., 6-month) duration of this study, signified by the fact that nearly half of the dropouts were attributed to consent withdrawal (25% and 18%, respectively, in the placebo and PEG 3350 groups), noncompliance (12% and 12%), and loss to follow-up (5% and 14%).

## 5. Conclusion

These post hoc analyses confirm that, when using modern, FDA-recommended efficacy endpoints for evaluation of laxative therapy, PEG 3350 is associated with robust 6-month efficacy and a favorable safety profile.

## Figures and Tables

**Table 1:** Demographics and baseline characteristics.

Outcome	PEG 3350 (*n* = 204)	Placebo (*n* = 100)	*P* value^†^
Age, mean (SD)	53.1 (14.9)	54.4 (15.0)	0.46
Sex, *n* (%)			
Female	175 (86)	83 (83)	
Male	29 (14)	17 (17)	0.56
Weight, mean (SD), kg	74.7 (16.3)	75.1 (15.6)	0.65
Duration of constipation, mean (SD)	23.4 (18.7)	22.6 (19.2)	0.66
Baseline^a^ CSBM/week, mean (SD)			
Week −2	0.7 (1.14)	0.6 (0.99)	
Week −1	0.8 (1.14)	0.9 (3.76)	
Baseline^a^ SBM/week, mean (SD)			
Week −2	3.2 (3.26)	3.4 (3.64)	
Week −1	2.9 (3.23)	4.0 (7.31)	

Abbreviations: CSBM, complete spontaneous bowel movement; PEG, polyethylene glycol; SBM, spontaneous bowel movement; SD, standard deviation ^∗^*P* < 0.05 (a) Fourteen-day pretreatment observation period ^†^should be *P* < 0.05.

**Table 2:** Change in BM-related symptoms per week over the 24-week study period.

BM-related symptom	Mean (SD) weekly change from baseline		*P* value
	PEG 3350 (*N* = 204)	Placebo (*N* = 100)	
Hard/lumpy^†^	–2.1 (3.06)	–0.9 (2.49)	**0.0014**
Straining^†^	–1.4 (3.63)	–0.8 (2.38)	0.0799
Cramping^‡^	–0.3 (0.62)	–0.1 (0.59)	**0.0272**
Gas^‡^	–0.1 (0.78)	–0.1 (0.62)	0.5949

Abbreviations: BM, bowel movement; PEG, polyethylene glycol; SD, standard deviation. †Mean change in the number of BMs/week with symptom. ‡Mean change in subject-assessed symptom score (0–4 scale).

**Table 3:** Gastrointestinal AEs (≥2% in any treatment group) during weeks 1 through 12 and weeks 13 through 24.

AE, *n* (%)	PEG 3350 (*N* = 204)	Placebo (*N* = 100)	All subjects (*N* = 304)	95% CI^†^	P value^‡^
*Weeks 1–12*					
Any GI AE	70 (34)	17 (17)	87 (29)	0.0748, 0.2714	**0.0018**
Diarrhea	28 (14)	6 (6)	34 (11)	0.0109, 0.1436	0.0525
Loose stools	16 (8)	1 (1)	17 (6)	0.0267, 0.1102	**0.0148**
Flatulence	13 (6)	2 (2)	15 (5)	0.0004, 0.087	0.1564
Abdominal distension	10 (5)	1 (1)	11 (4)	0.0035, 0.0745	0.1090
Nausea	9 (4)	1 (1)	10 (3)	–15 E-5, 0.0684	0.1742
Abdominal pain	2 (1)	2 (2)	4 (1)	–0.041, 0.0204	0.6004
Dyspepsia	1 (0)	2 (2)	3 (1)	–0.044, 0.014	0.2527

*Weeks 13–24*					
Any GI AE	19 (9)	12 (12)	31 (10)	–0.102, 0.0483	0.5455
Diarrhea	12 (6)	7 (7)	19 (6)	–0.071, 0.0483	0.8016
Loose stools	0	2 (2)	2 (1)	–0.047, 0.0074	0.1075

Abbreviations: AE, adverse event; CI, confidence interval; GI, gastrointestinal; PEG, polyethylene glycol. ^†^95% CI for difference in proportion. ^‡^Two-tailed Fisher's exact test.

## Data Availability

The data that support the findings of this study can be obtained from the corresponding author upon request. The data have not been made publicly available so as to protect intellectual property.

## Supplementary Materials

Supplementary Materials The supplementary material includes details on patient disposition in the study outcomes and a summary of clinical responses with other therapies.
