# Supplementary material for: Polyethylene Glycol 3350 in the Treatment of Chronic Idiopathic Constipation: Post hoc Analysis Using FDA Endpoints
Source: Can J Gastroenterol Hepatol. 2022 Sep 9;2022:3533504. doi: 10.1155/2022/3533504 (PMC9481403; doi:10.1155/2022/3533504)
Supplement: Supplementary Materials — The supplementary material includes details on patient disposition in the study outcomes and a summary of clinical responses with other therapies. [file 3533504.f1.zip › 3533504.f1/Patient Disposition_BM Outcomes_Response with Other Therapies.docx]

**Supplemental Material**

**Supplemental Figure 1.** Patient Disposition

Abbreviation: PEG, polyethylene glycol.

**Supplemental Table 1.** CSBM and SBM Outcomes

| **Outcome** | **PEG 3350 (n = 204)** | **Placebo (n = 100)** | ***P* value^a^** |
| --- | --- | --- | --- |
| **CSBM** |  |  |  |
| Weekly CSBM response for 9/12 weeks^b^ | 42% | 13% | **< .0001** |
| Weekly CSBM response for 18/24 weeks | 43% | 11% | **< .0001** |
| Weekly CSBM response for 6/12 weeks | 60% | 26% | **< .0001** |
| Weekly CSBM response for 12/24 weeks | 54% | 21% | **< .0001** |
| Continuous CSBM response^c^ | 35% | 8% | **< .0001** |
| **SBM** |  |  |  |
| Weekly SBM response for 9/12 weeks | 49% | 16% | **< .0001** |
| Weekly SBM response for 18/24 weeks | 48% | 13% | **< .0001** |
| Weekly SBM response for 6/12 weeks | 66% | 33% | **< .0001** |
| Weekly SBM response for 12/24 weeks | 60% | 23% | **< .0001** |
| Continuous SMB response^c^ | 40% | 9% | **< .0001** |

Abbreviations: CIC, chronic idiopathic constipation; CMH, Cochran-Mantel-Haenszel; CSBM, complete spontaneous bowel movement; FDA, US Food and Drug Administration; PEG, polyethylene glycol; SBM, spontaneous bowel movement.

Weekly responder: ≥3 CSBMs/SBMs and an increase of ≥1 CSBMs/SBMs from baseline, and data are available on at least 4 days in a respective week.

^a^ CMH chi-square test, adjusting for pooled study sites, to ensure homogeneity/consistency across the treatment arms.

^b^ FDA CIC endpoint: weekly responder for ≥9 weeks out of the 12-week treatment period including 3 of the last 4 weeks of the period.

^c^ Weekly responder for ≥9 out first 12 weeks and ≥9 out of last 12 weeks.

**Supplemental Table 2.** Randomized Controlled Trials Evaluating Effect of Treatment in Patients With Constipation Using FDA-Recommended Criteria[[12-18](data:text/html,%20Lembo%202011;%20Schoenfeld%202018;%20Lacy%202015;%20Miner%20Am%20J%20Gastro%202017;%20DeMicco%202017;%20Yiannakou%202015;%20Piessevaux%202015)]

| **Study** | **No. of patients** | **Regimen** | **Baseline CSBM/week** | **Response definition** | **Results (% responders)** |
| --- | --- | --- | --- | --- | --- |
| **Linaclotide** | | | | | |
| Lembo 2012 | Trial 303  LIN 145 μg: 217  LIN 290 μg: 216  PLA: 209  Trial 01  LIN 145 μg: 213  LIN 290 μg: 202  PLA: 215 | LIN 145 μg QD  LIN 290 μg QD  PLA QD (both trials) | Trial 303  LIN 145 μg: 0.3  LIN 290 μg: 0.2  PLA: 0.3  Trial 01  LIN 145 μg: 0.3  LIN 290 μg: 0.3  PLA: 0.3 | ≥3 CSBMs/wk and ↑ of ≥1 CSBM/wk from baseline for 9/12 weeks | Trial 303  LIN 145 μg: 21.2% (*P* < .01^a^)  LIN 290 μg: 19.4% (*P* < .01^a^)  PLA: 3.3%  Trial 01  LIN 145 μg: 16.0% (*P* < .01^a^)  LIN 290 μg: 21.3% (*P* < .001^a^)  PLA: 6.0% |
| Schoenfeld 2018 | LIN 72 μg: 411  LIN 145 μg: 411  PLA: 401 | LIN 72 μg QD  LIN 145 μg QD  PLA QD | LIN 72 μg: 0.2  LIN 145 μg: 0.2  PLA: 0.3 | ≥3 CSBMs/wk and ↑ of ≥1 CSBM/wk from baseline for 9/12 weeks | LIN 72 μg: 13.4% (*P* < .001^a^)  LIN 145 μg: 12.4% (*P* < .001^a^)  PLA: 4.7% |
| Lacy 2015 | LIN 145 μg: 153  LIN 290 μg: 159  PLA: 171 | LIN 145 μg QD  LIN 290 μg QD  PLA QD | LIN 145 μg: 0.2  LIN 290 μg: 0.2  PLA: 0.2 | ≥3 CSBMs/wk and ↑ of ≥1 CSBM/wk from baseline for 9/12 weeks | LIN 145 μg: 15.7% (*P* < .05^a^)  LIN 290 μg: 16.4% (*P* < .05^a^)  PLA: 7.6% |
| **Plecanatide** | | | | | |
| Miner 2017 | PLE 3 mg: 452  PLE 6 mg: 441  PLA: 452 | PLE 3 mg QD  PLE 6 mg QD  PLA QD | PLE 3 mg: 0.3  PLE 6 mg: 0.3  PLA: 0.4 | ≥3 CSBMs/wk and ↑ of ≥1 CSBM/wk from baseline for 9/12 wks and 3 of last 4 wks | PLE 3 mg: 21.0% (*P* < .001^a^)  PLE 6 mg: 19.5% (*P* < .001^a^)  PLA: 10.2% |
| DeMicco 2017 | PLE 3 mg: 452  PLE 6 mg: 441  PLA: 452 | PLE 3 mg QD  PLE 6 mg QD  PLA QD | PLE 3 mg: 0.3  PLE 6 mg: 0.3  PLA: 0.3 | ≥3 CSBMs/wk and ↑ of ≥1 CSBM/wk from baseline for 9/12 wks and 3 of last 4 wks | PLE 3 mg: 20.2% (*P* < .01^a^)  PLE 6 mg: 20.0% (*P* < .01^a^)  PLA: 12.8% |
| **Prucalopride** | | | | | |
| Yiannakou 2015 | PRU 2 mg: 184  PLA: 186 | PRU 2 mg QD  PLA QD | PRU 2mg: NR  PLA: NR | Mean weekly frequency of ≥3 CSBMs/wk over 12 wks | PRU 2 mg: 37.9% (*P* < .0001^a^)  PLA: 17.7% |
| Piesseavaux 2015 | PRU 2 mg: 182  PLA: 182 | PRU 2 mg QD  PLA QD | PRU 2mg: NR  PLA: NR | Mean weekly frequency of ≥3 CSBMs/wk over 24 wks | PRU 2 mg: 25.1% (NSD)  PLA: 20.7% |

Abbreviations: CSBM, complete spontaneous bowel movement; LIN, linaclotide; NR, not reported; NSD, not significantly different vs placebo; PLA, placebo; PLE, plecanatide; PRU, prucalopride; QD, once daily; wk, week.

^a^ Versus placebo.
