# Supplementary figures and images for: Polyethylene Glycol 3350 in the Treatment of Chronic Idiopathic Constipation: Post hoc Analysis Using FDA Endpoints
Source: Can J Gastroenterol Hepatol. 2022 Sep 9;2022:3533504. doi: 10.1155/2022/3533504 (PMC9481403; doi:10.1155/2022/3533504)

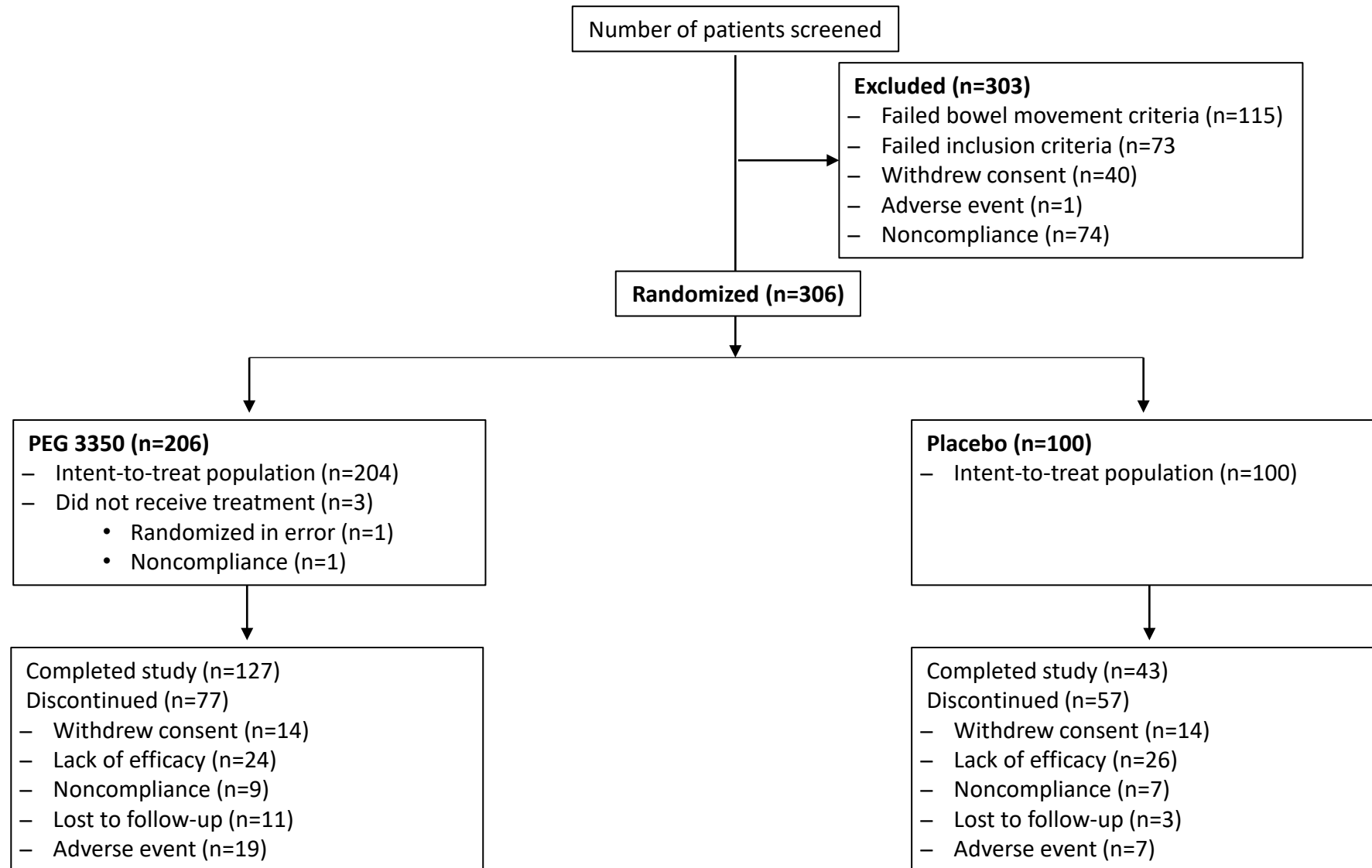

Supplement: Supplementary Materials — The supplementary material includes details on patient disposition in the study outcomes and a summary of clinical responses with other therapies. [file 3533504.f1.zip › 3533504.f1/Supplementary Fig 1_Patient Disposition.pdf]
